# Supplementary material for: Wound Healing Efficacy of Cucurbitaceae Seed Oils in Rats: Comprehensive Phytochemical, Pharmacological, and Histological Studies Tackling AGE/RAGE and Nrf2/Ho-1 Cue
Source: Pharmaceuticals (Basel). 2024 Jun 5;17(6):733. doi: 10.3390/ph17060733 (PMC11206300; doi:10.3390/ph17060733)
Supplement: Supplementary file 1 [file pharmaceuticals-17-00733-s001.zip › pharmaceuticals-3006367-supplementary.pdf]

pharmaceuticals

MDPI

Table S1: p-values of Levene's & Shapiro-Wilk tests

Test	AGE		RAGE	
	**p-value**	**significant**	**p-value**	**significant**
**Levene's test**	p=0.5328	ns	p = 0.6066	ns
**Shapiro-Wilk**				
Wound injury	0.2530	Pass normality	0.4633	Pass normality
Wound injury+PSO	0.6369		0.4043	
Wound injury+HSO	0.3884		0.3480	
Wound injury+CSO	0.4633		0.7262	
Wound injury+ZSO	0.6933		0.8428	

Abbreviations: AGE: the advanced glycation end products, RAGE: receptor of advanced glycation end products, ns: non-significant.
